# The neural correlates of mental arithmetic in adolescents: a longitudinal fNIRS study

**DOI:** 10.1186/s12993-018-0137-8

**Published:** 2018-03-10

**Authors:** Christina Artemenko, Mojtaba Soltanlou, Ann-Christine Ehlis, Hans-Christoph Nuerk, Thomas Dresler

**Affiliations:** 1 LEAD Graduate School & Research Network, University of Tuebingen, Tuebingen, Germany; 2 Department of Psychology, University of Tuebingen, Tuebingen, Germany; 3 Graduate Training Centre of Neuroscience/IMPRS for Cognitive and Systems Neuroscience, Tuebingen, Germany; 4 Leibniz-Institut für Wissensmedien, Tuebingen, Germany; 5 Department of Psychiatry and Psychotherapy, University of Tuebingen, Tuebingen, Germany

**Keywords:** Functional near-infrared spectroscopy (fNIRS), Adolescents, Mental arithmetic, Arithmetic complexity, Longitudinal development, Natural setting

## Abstract

**Background:**

Arithmetic processing in adults is known to rely on a frontal-parietal network. However, neurocognitive research focusing on the neural and behavioral correlates of arithmetic development has been scarce, even though the acquisition of arithmetic skills is accompanied by changes within the fronto-parietal network of the developing brain. Furthermore, experimental procedures are typically adjusted to constraints of functional magnetic resonance imaging, which may not reflect natural settings in which children and adolescents actually perform arithmetic. Therefore, we investigated the longitudinal neurocognitive development of processes involved in performing the four basic arithmetic operations in 19 adolescents. By using functional near-infrared spectroscopy, we were able to use an ecologically valid task, i.e., a written production paradigm.

**Results:**

A common pattern of activation in the bilateral fronto-parietal network for arithmetic processing was found for all basic arithmetic operations. Moreover, evidence was obtained for decreasing activation during subtraction over the course of 1 year in middle and inferior frontal gyri, and increased activation during addition and multiplication in angular and middle temporal gyri. In the self-paced block design, parietal activation in multiplication and left angular and temporal activation in addition were observed to be higher for simple than for complex blocks, reflecting an inverse effect of arithmetic complexity.

**Conclusions:**

In general, the findings suggest that the brain network for arithmetic processing is already established in 12–14 year-old adolescents, but still undergoes developmental changes.

**Electronic supplementary material:**

The online version of this article (10.1186/s12993-018-0137-8) contains supplementary material, which is available to authorized users.

## Background

Arithmetic processing consistently activates a fronto-parietal network in the adult brain, which includes parietal regions such as the superior parietal lobule (SPL) and inferior parietal lobule (IPL), and frontal regions such as the inferior frontal gyrus (IFG), middle frontal gyrus (MFG) and left superior frontal gyrus (for a meta-analysis see [1]; see also [2–4]). In children, arithmetic processing also generally recruits a similar fronto-parietal network ([5–7]; for a review see [8]); however, some differences have been reported between children and adults. Specifically, arithmetic processing seems to be less functionally specialized in children, which leads to less parietal activation especially in the intraparietal sulcus (IPS) for children compared to adolescents and for adolescents compared to adults [7, 9]. But since neurocognitive development does not necessarily progress linearly, it is not possible to generalize the neural and behavioral correlates of arithmetic processing from either adults or children to adolescents, an underrepresented age cohort in neurocognitive research (for a review see [8]; for a meta-analysis see [10]). Therefore, the focus of the current study is to systematically investigate the neurocognitive correlates of arithmetic processing in adolescents by considering developmental activation changes as well as different complexity levels in all basic arithmetic operations.

### Neurocognitive development of arithmetic processing in children and adolescents

During childhood, the neurocognitive underpinnings of arithmetic change with age and development: with increasing age, children show decreasing activation in bilateral SFG, MFG and the left IFG, indicating reduced reliance on working memory and attentional resources, and simultaneously increasing activation in the left supramarginal gyrus and IPS, which is a core area in number processing ([11]; see also [7, 9, 12, 13]; for meta-analyses see [14, 15]). This so-called *fronto*-*parietal shift* in brain activation can be considered to represent the increasing functional specialization of the parietal cortex for arithmetic processing accompanied by decreasing reliance on domain-general cognitive processes [16].

While there is broad evidence for the fronto-parietal shift during development, the specific activation changes seem to depend on age, arithmetic content and design. For instance, contradictory findings exist from cross-sectional to longitudinal studies on arithmetic development in 7–9 year-old children within 1 school year: Rosenberg-Lee et al. [17] found increased fronto-parietal activation in a cross-sectional study, while Qin et al. [18] found a general reduction of activation in the fronto-parietal network and increasing hippocampal activation in a longitudinal study.

In summary, there is evidence for the fronto-parietal activation shift during development in general. However, contradictory neural findings were reported for development during elementary school when arithmetic skills are taught, and furthermore children’s arithmetic development is quite heterogeneous [15, 19], which limits the conclusions of cross-sectional designs. Finally, very little is known about arithmetic development in adolescents during secondary school when they already possess arithmetic knowledge. Therefore, we chose to investigate the neurocognitive underpinnings of arithmetic development during 1 year of secondary school in a longitudinal design.

### Neurocognitive processing of arithmetic complexity in children

Besides interindividual differences in arithmetic skills during development, neurocognitive processing is also affected by arithmetic complexity which differs between problems (for an overview see [20, 21]). In mental arithmetic, complexity is increased when additional arithmetic procedures have to be applied (e.g., carry and borrow procedures) or when the numerical magnitude of the operands is relatively large (e.g., two-digit versus single-digit operands). Problems that require carrying in addition (unit sum is larger than 9) or borrowing in subtraction (subtrahend unit is larger than minuend unit) are more difficult for children than problems without these procedures (e.g., [22]). The carry and borrow effects are primarily associated with activation in frontal areas such as the left IFG, bilateral MFG and SFG, as well as with activation in parietal areas such as the left IPS in adults [23–27]. However, the neural correlates of the carry and borrow effects have so far not been investigated in children or adolescents.

Arithmetic complexity indexes not only the need for additional arithmetic procedures like carrying or borrowing, which recruit mainly domain-general processes in children [28], but also domain-specific attributes such as numerical magnitude. Indeed, arithmetic problems with relatively large operands are more difficult to solve than problems with relatively small operands as reflected by the problem size effect in children (e.g., [29]). On a neural level, the problem size effect in single-digit arithmetic in children was found to be associated with increased activation in the IPS, SPL, left MFG, and IFG in addition [17, 30, 31] and subtraction [30, 32], and in the left IPS and left DLPFC in multiplication [32]. Moreover, two-digit (as compared to single-digit) problems led to higher activation in IPS and angular gyrus (AG) in addition [5] and in the right MFG in multiplication [33]. Furthermore, with increasing problem size less activation in AG and superior temporal gyri was observed [5, 30].

To summarize, the literature suggests that carrying/borrowing and problem size both increase arithmetic complexity by enhancing domain-general and domain-specific processing demands, respectively. Although both types of arithmetic complexity are associated with distributed fronto-parietal activation, we expect the carry and borrow effects to be represented mostly in frontal and partially in parietal brain regions in children, while the opposite pattern is expected for the problem size effect. In this study, we will investigate both types of arithmetic complexity in different arithmetic operations, since brain activation patterns have been shown to be operation-specific, particularly in children and adolescents ([30, 32, 34]; but see [6]) and the effects of arithmetic complexity seem to depend on the operation (e.g., for multiplication see [35, 36]). The neural substrates of division have not been investigated in children and adolescents so far (except for a structural diffusion tensor imaging (DTI) study [37]). Therefore, arithmetic complexity will be addressed using all four basic arithmetic operations in the current study, i.e., the carry and borrow effects in addition and subtraction, and the problem size effect in multiplication and division.

### An ecologically valid assessment of arithmetic processing in children

The typical arithmetic tasks used in neuroimaging studies differ from the tasks usually employed in schools, where children and adolescents typically have to *produce* written solutions for arithmetic problems, often in a time-restricted manner. Typical neuroimaging studies investigating the correlates of arithmetic in adults use verification or forced choice paradigms instead of written production paradigms (as an exception see e.g., [38] for an oral production paradigm) and fixed designs instead of self-paced designs (as an exception see e.g., [39] for a self-paced design). Importantly, these differences need to be considered, because they seem to evoke different cognitive processes as well.

First, verification and forced choice paradigms, which are particularly apt for movement-sensitive fMRI measurements, allow for shortcut strategies depending on the distractor, and include additional decision and recognition processes (cf. [25, 40–42]) not involved in the spontaneous calculation of arithmetic problems. Thus, these paradigms have little in common with the written production process usually employed in school. Second, in fixed designs, the average neural activation across trials is compared between conditions. If the reaction time differs between conditions, this can lead to systematic activation differences depending on task duration, while self-paced responses allow for a different number of trials within each block. Self-paced designs were further shown to be comparable to fixed designs in sensitivity and even superior in reproducibility and reliability [43].

In order to assess the neural activation patterns underlying calculation in a more natural setting, a written production paradigm within a self-paced block design was used in this study (cf. [33]). A written production paradigm as used in schools can be realized by using functional near-infrared spectroscopy (fNIRS) to assess task-related neural activation, since this method is relatively movement-insensitive, allows for natural body postures, and can be easily administered in students [44–46].

### Objectives

The aim of this study is to understand arithmetic development and complexity in adolescents, by investigating arithmetic processing for all four basic operations with varying complexity in a natural setting, in a longitudinal design so that individual differences can be controlled. Specifically, we will address the following issues:

1. Do brain activity patterns change within frontal and parietal regions for all arithmetic operations over the course of 1 year? In line with the frontal-to-parietal shift hypothesis, a simultaneous decrease in frontal activation and increase in parietal activation is expected.
2. Do the neural correlates of the basic arithmetic operations in adolescents vary with arithmetic complexity? It is expected that the carry effect in addition and the borrow effect in subtraction mainly lead to larger frontal activation, while problem size effects in multiplication and division mainly lead to larger parietal activation associated with number magnitude processing.

These questions will be addressed by investigating all four basic arithmetic operations at differing complexity levels in 12–14 year-old adolescents in grades 6 and 7 in an fNIRS study. While basic arithmetic skills are mostly acquired in elementary school, it is unclear whether they still undergo further automatization in older children and adolescents. In order to assess arithmetic processing in a natural setting, a written production paradigm with self-paced responses will be used, and a standardized arithmetic test will serve for the evaluation of generalizability to arithmetic skills.

## Methods

### Participants

Twenty-six adolescents (20 male) were recruited through local schools and a university mailing list. Nineteen adolescents completed both measurements at the end of grades 6 and 7 (16 male; age in grade 6: *M* ± SD = 12.19 ± 0.33, range = 11.75–12.75 years; age in grade 7: *M* ± SD = 13.34 ± 0.35, range = 12.75–13.92 years). The time interval between the two measurements was 1 year (interval in months: *M* ± SD = 13.73 ± 1.05, range = 12–15 months). All adolescents attended a German secondary school, showed no history of neurological or mental disorders, and all except for three left-handed participants were right-handed. Non-verbal intelligence was assessed by the matrix reasoning subtest of HAWIK-IV and verbal intelligence by the similarities subtest of HAWIK-IV (Hamburg–Wechsler-Intelligenztest für Kinder-IV; [47]). The adolescents showed on average scores of 108.68 ± 10.91 for non-verbal intelligence and 110.00 ± 9.57 for verbal intelligence (IQ ± SD). The standardized assessed arithmetic ability of the adolescents was 57.21 ± 8.70 (T score ± SD), as assessed by the arithmetic subtest of DEMAT 5 + (Deutscher Mathematiktest für fünfte Klassen; [48]).

### Arithmetic tasks

All adolescents solved computerized addition, subtraction, multiplication, and division tasks which were presented in blocks with two complexity levels (simple, complex). The addition and subtraction tasks consisted of two two-digit operands with a two-digit solution. In simple blocks, the addition problems did not require the carry procedure and, accordingly, the subtraction problems did not require the borrow procedure. In complex blocks, the arithmetic problems requiring and not requiring the carry and borrow procedure were mixed, with no more than two no-carry or no-borrow problems in a row. The mixing of problems with and without carry or borrow procedure was meant to ensure that the adolescents had to decide whether or not to apply the carry or borrow procedure in each problem, and not just apply it during the whole block. The subtraction problems were constructed as the inverse of addition problems (e.g., 26 + 52 = 78 → 78 − 52 = 26). In the multiplication task, simple blocks included problems with two single-digit operands between 2 and 9 (solutions range between 12 and 72), while complex blocks included problems with one single-digit operand between 2 and 9 and one two-digit operand between 12 and 19 (solutions range between 32 and 162). The division problems were constructed as the inverse of multiplication problems (e.g., 8 × 4 = 32 → 32 : 4 = 8).

The items were randomly chosen from a set of 50 items per operation and complexity level. In each stimulus set, the operands were matched in their numerical magnitude and parity. The units and decades of the operands were matched in their numerical magnitude and the position of the larger operand was counterbalanced in each condition. Pure decades (e.g., 20), tie numbers (e.g., 44) and numbers sharing the same digit between operands or the solution (e.g., 34 + 38) were excluded (for a similar procedure see [25]).

**Fig. 1:**
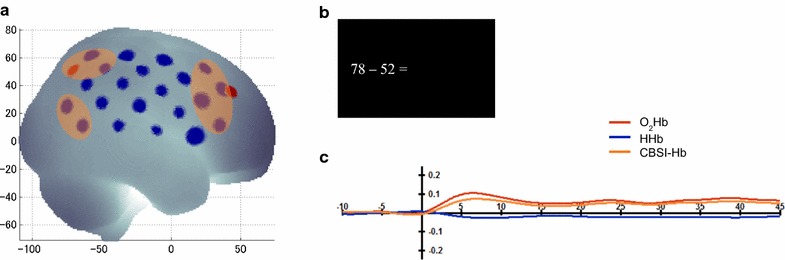
**a** Positions of the fNIRS channels mapped on the brain (by Minako Uga). As an example, the right brain hemisphere is shown along with the red marked positions for the orientation of the probeset (the same applies to the left hemisphere). Channels included in the parietal, frontal, and parieto-temporal ROIs are marked by the orange boxes. **b** Example trial for the arithmetic task. The adolescents had to produce the correct solution and write it on the screen. **c** Exemplary course of the fNIRS signal. The block average curves of O_2_Hb (red), HHb (blue) and signal corrected by CBSI (orange) are given for the left parietal ROI for simple subtraction in grade 7

The arithmetic tasks were presented in a block design with a block length of 45 s, an inter-block-interval of 20 s, and four blocks per operation and complexity level, i.e., 32 blocks in total. Simple and complex blocks of all operations were pseudorandomized for each participant and presented in the same order at both measurement points. The arithmetic problems along with an equal sign were horizontally presented in white against black background on the left side of the computer screen (cf. Fig. 1b) using the software package Presentation (NeuroBehavioral Systems, Inc., Berkeley, USA). In a production paradigm, the adolescents were instructed to mentally solve the arithmetic problems and to write the solution on the touch screen using a contact pen (cf. [33]). The trace of the contact pen on the touch screen during the written responses was not visible to the adolescents and the screen remained black in order to emphasize mental arithmetic. Within a self-paced design, each trial followed after a fixed inter-trial-interval of 500 ms, after the button press or after the response time limit if no response was made. Therefore, the number of items within a block varied within and between participants. The time limits were chosen based on the study of Huber et al. [49], i.e., calculated by *M* + 1 SD regarding the sum of correctly and incorrectly solved trials within the time window: 5 and 6 s for simple and complex addition (a minimum of 9 simple and 8 complex trials per block), 6 and 9 s for simple and complex subtraction (a minimum of 8 simple and 5 complex trials per block), 7 and 25 s for simple and complex multiplication (a minimum of 7 simple and 2 complex trials per block), and 7 and 45 s for simple and complex division (a minimum of 7 simple and 1 complex trial per block), respectively. The testing phase was preceded by four practice trials for adolescents to become familiar with the arithmetic tasks. In total, the arithmetic tasks lasted 35 min and additionally included a break after two out of four runs.

### Procedure

The measurements were conducted with adolescents at the end of grade 6 and 1 year later at the end of grade 7. In both experimental sessions, the adolescents solved computerized tasks for all four basic arithmetic operations during fNIRS recording in a dimly lit room. The adolescents were seated in front of the touch screen, which was placed in an angle of 37 degrees, and the fNIRS optodes were inserted into the cap on their head after pushing aside the hair at each position (for a picture of the general experimental setup, see Fig. 1c in the study of [33], p. 727). Additionally, arithmetic ability and intelligence were assessed in grade 6 before and after fNIRS measurement, respectively. The adolescents underwent further assessments in each session and performed two additional tasks during the fNIRS recording in grade 6 that are not part of this study.

### fNIRS data acquisition

fNIRS was measured using the ETG-4000 Optical Topography System (Hitachi Medical Corporation, Tokyo, Japan). Continuous laser diodes with wavelengths of 695 ± 20 nm and 830 ± 20 nm were used as light sources. The sampling rate was 10 Hz. Two probesets with 22 channels and an inter-optode distance of 30 mm were integrated in an elastic cap in order to cover the left and right hemisphere. The probesets were placed into the cap by localizing the upper channels in the back at P3/P4 and orienting this channel row towards F3/F4 (according to the 10/20 system [50]; cf. Fig. 1a). Note that because of the constant optode distance, the brain areas underlying the channels varied depending on cap size (54, 56, 58 cm).

### Analysis of behavioral data

As a behavioral measure in a self-paced design, the number of presented trials (i.e., the sum of presented trials during all experimental blocks of a certain condition) was calculated. Furthermore, the written solutions of the subjects were visualized with the help of a RON (ReadOut Numbers) program (Ploner, 2014) and manually analyzed for correctness. Response times (RT) were defined from stimulus onset to the final button press when the subject had finished producing the solution to the arithmetic problem. For RT analysis, only RTs of correct trials were included and the median RT was calculated for each subject and condition. Accuracy (ACC) was calculated by the proportion of correctly solved trials. The behavioral data was statistically analyzed by a repeated-measures ANOVA with the within-subject factors grade (6, 7) and complexity (simple, complex) for each task.^1^

### fNIRS data analysis

For each fNIRS channel, the optical data for the two wavelengths were transformed into relative concentration changes of oxygenated (O_2_Hb) and deoxygenated haemoglobin (HHb). The analysis of the fNIRS data was conducted using custom MATLAB (The MathWorks, Inc., USA) scripts. For data preprocessing, a bandpass filter of 0.01–0.2 Hz was applied to the data [51]. In the next step, noisy fNIRS channels were interpolated by neighboring channels, and blocks containing uncorrectable artifacts were excluded from the analysis. Moreover, to deal with remaining motion artifacts, the signal was corrected by correlation-based signal improvement (CBSI) according to Cui et al. [52]. CBSI corrects the signal based on the assumption that simultaneous increases in O_2_Hb and decreases in HHb are indicators of cortical activation [53] and is among the best methods for reducing motion artifacts [54], particularly in children and adolescents. Afterwards, the 45 s blocks were averaged across the four repetitions for each condition and baseline-corrected mean amplitudes of the hemodynamic responses were calculated channel-wise for each participant (baseline: 10 s).

Based on virtual registration [55–57] and according to the automated anatomic labeling (AAL) atlas [58], regions of interest (ROIs) were defined for left and right parietal areas (including SPL and IPL), frontal areas (including MFG and IFG), and parieto-temporal areas (including AG and middle temporal gyrus (MTG); cf. Fig. 1a for the position of the ROIs and Fig. 1c for an example signal within a ROI). Based on the individual activation peak within each ROI (cf. [59]), the contrasts for each grade and complexity level were calculated and the significance of activation was tested for each task using one-sample *t*-tests against zero (significance level of .05, False Discovery Rate (FDR) corrected for multiple statistical comparisons, cf. [60]). The main analysis focuses on the developmental fronto-parietal shift hypothesis and therefore was performed on the frontal and parietal ROIs within a 2 grade (6, 7) × 2 complexity (simple, complex) × 2 ROI (frontal, parietal) × 2 hemisphere (left, right) repeated measures ANOVA for each task. Additionally, since some studies found temporal and AG activation to be associated with arithmetic during development (e.g., [32]), another analysis was performed on the parieto-temporal ROIs within a 2 grade (6, 7) × 2 complexity (simple, complex) × 2 hemisphere (left, right) repeated measures ANOVA for each task.

For significant effects of grade and arithmetic complexity, separate ANCOVAs were conducted by including the difference in the number of presented trials for the respective effect as a covariate. This procedure was conducted based on the self-paced design in accordance with Soltanlou et al. [33], but the results should be taken with caution, because the effect of the covariate was not independent from the investigated effects (see [61]). Furthermore, brain-behavior-correlations between the number of presented trials and cortical activation in each ROI were calculated for the effect of grade (grade 7 vs. grade 6) and the effect of arithmetic complexity (complex vs. simple) for each task. Results and discussion of the brain-behavior-correlations are reported in Additional file 1.

## Results

### Behavioral data

**Fig. 2:**
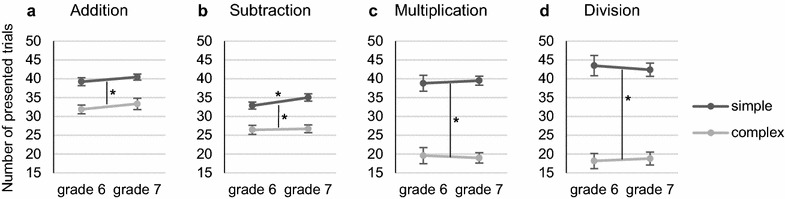
Number of presented trials in the **a** addition, **b** subtraction, **c** multiplication, and **d** division tasks. Significant arithmetic complexity and grade effects are marked (*p < .05). Error bars indicate 1 SE of *M*

In general, better behavioral performance means that the adolescents solved more problems during the blocks (larger number of presented trials), were faster in solving the problems (lower RT), and made fewer errors (larger ACC). Behavioral data was analyzed by a 2 grade (6, 7) × 2 complexity (simple, complex) ANOVA for each task (for statistical details see Table 1, see also Fig. 2).

**Table 1:** Behavioral results for the arithmetic tasks

Task	N_presented trials_			RT			ACC		
	*F*_1,18_	*p*	$\eta_{p}^{2}$	*F*_1,17_	*p*	$\eta_{p}^{2}$	*F*_1,17_	*p*	$\eta_{p}^{2}$
Addition									
Grade	2.93	.104	.140	1.49	.283	.081	3.98	.062	.190
Complexity	*166.33*	*< .001*	*.902*	*92.72*	*< .001*	*.845*	*15.63*	*.001*	*.479*
Grade × complexity	0.08	.783	.004	1.19	.291	.065	*7.75*	*.013*	*.313*
Subtraction									
Grade	1.61	.220	.082	1.43	.263	.073	*7.32*	*.015*	*.301*
Complexity	*299.87*	*< .001*	*.943*	*178.29*	*< .001*	*.913*	*7.20*	*.016*	*.297*
Grade × complexity	*6.61*	*.019*	*.269*	0.39	.539	.023	0.03	.877	.001
Multiplication									
Grade	0.00	.984	.000	0.73	.405	.041	3.24	.090	.160
Complexity	*191.66*	*< .001*	*.914*	*101.87*	*< .001*	*.857*	*55.25*	*< .001*	*.765*
Grade × complexity	1.27	.275	.066	0.24	.634	.014	0.16	.698	.009
Division									
Grade	0.03	.860	.002	0.69	.418	.039	1.15	.298	.063
Complexity	*150.03*	*< .001*	*.893*	*24.79*	*< .001*	*.593*	*67.90*	*< .001*	*.800*
Grade × complexity	0.47	.500	.026	0.58	.458	.033	0.66	.428	.037

Significant effects are shown in italics.

In addition, there was a significant main effect of complexity for all measures, indicating a better performance on simple problems. Furthermore, a significant interaction of grade and complexity for ACC indicated that the adolescents were making fewer errors on simple problems from grade 6 to grade 7 (post hoc test: *p* = .015). No other effects were significant.

In subtraction, the main effect of grade was significant for ACC, indicating that the adolescents made fewer errors from grade 6 to grade 7. There was a significant main effect of complexity for all measures, indicating a better performance on simple problems. Furthermore, a significant interaction of grade and complexity for the number of presented trials indicated that the adolescents were only solving more simple problems from grade 6 to grade 7 (post hoc test: *p* = .036), which corresponds to the addition result for ACC.

In multiplication, there was a significant main effect of complexity for all measures, indicating a better performance for simple problems. No other effects were significant.

In division, there was a significant main effect of complexity for all measures, indicating a better performance for simple problems. No other effects were significant.

The standardized assessed arithmetic ability correlated positively with behavioral performance for all arithmetic tasks (average number of presented trials) in grade 6 (*r* = .504, *p* = .028) and showed a similar trend for grade 7 (*r* = .443, *p* = .058), indicating that arithmetic performance measured in the experimental task resembles the arithmetic skill of the adolescents.

### fNIRS data

Cortical activation within the ROIs defined above was analyzed separately for complexity and grade level for each task (cf. Additional file 1: Figs. S1–S4). In all arithmetic tasks, significant activation was found in the bilateral parietal and bilateral frontal areas for simple and complex arithmetic in both grade levels (*t*s(18) > 2.50, FDR-corrected *p*s < .05). Additionally, significant deactivation was found in the left parieto-temporal area for complex addition in grade 6 (*t*(18) = − 2.32, FDR-corrected *p* < .05; cf. Additional file 1: Fig. S1). There were no other areas with significant activation or deactivation (all FDR-corrected *p*s > .05).

#### Activation in MFG/IFG and SPL/IPL

First to examine our main question regarding the fronto-parietal shift in brain activation and arithmetic complexity effects in frontal (MFG/IFG) and parietal (SPL/IPL) brain regions, a 2 grade (6, 7) × 2 complexity (simple, complex) × 2 ROI (frontal, parietal) × 2 hemisphere (left, right) ANOVA was calculated for each task.

In addition, no significant effects were observed (all *p*s > .1).

**Fig. 3:**
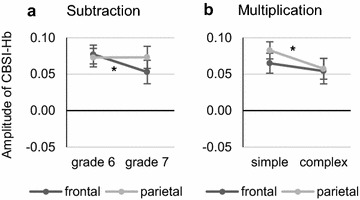
Cortical activation in the frontal and parietal ROIs. **a** Significant reduction in frontal activation from grade 6 to grade 7 in the subtraction task (*p < .05). **b** Significantly increased parietal activation for simple compared to complex blocks in the multiplication task (*p < .05). Error bars indicate 1 SE of *M*

In subtraction, there was a significant main effect of hemisphere (*F*(1, 18) = 7.13, *p* = .016, $\eta_{p}^{2}$ = .284) indicating that activation was larger in the left hemisphere than in the right hemisphere. Furthermore, the interaction of grade and ROI was significant (*F*(1, 18) = 4.44, *p* = .050, $\eta_{p}^{2}$ = .198) and a one-sided post hoc test based on our directed hypothesis revealed that only frontal activation decreased from grade 6 to grade 7 (*p* = .046; cf. Fig. 3a). No other effects were significant (all *p*s > .1).

In multiplication, there was a significant main effect of hemisphere (*F*(1, 18) = 7.23, *p* = .015, $\eta_{p}^{2}$ = .287) indicating that activation was larger on the left hemisphere than on the right hemisphere. Furthermore, a significant main effect of complexity (*F*(1, 18) = 5.20, *p* = .035, $\eta_{p}^{2}$ = .224) and a significant interaction of complexity and ROI (*F*(1, 18) = 5.74, *p* = .028, $\eta_{p}^{2}$ = .242) were found, indicating that only parietal activation was increased for simple compared to complex problems (post hoc test: *p* = .012; cf. Fig. 3b). No other effects were significant (all *p*s > .05).

In division, there was a significant main effect of hemisphere (*F*(1, 18) = 4.50, *p* = .048, $\eta_{p}^{2}$ = .200) and a significant interaction of ROI and hemisphere (*F*(1, 18) = 12.15, *p* = .003, $\eta_{p}^{2}$ = .403) indicating that only parietal activation is larger on the left hemisphere than on the right hemisphere (*p* = .005). No other effects were significant (all *p*s > .05).

#### Activation in AG/MTG

**Fig. 4:**
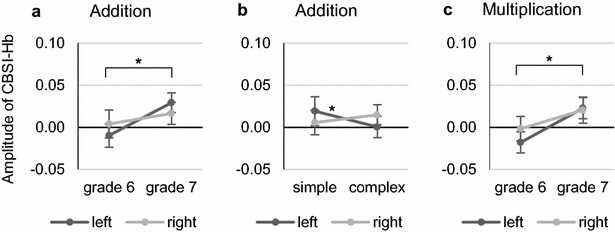
Cortical activation in the parieto-temporal ROIs. **a** Significant change in parieto-temporal activation from grade 6 to grade 7 in the addition task (*p < .05). **b** Significantly increased left parieto-temporal activation for simple compared to complex blocks in the addition task (*p < .05). **c** Significant change in parieto-temporal activation from grade 6 to grade 7 in the multiplication task (*p < .05). Error bars indicate 1 SE of *M*

Next to investigate developmental activation changes and arithmetic complexity effects for parieto-temporal brain regions (AG/MTG), an additional analysis was performed for parieto-temporal activation within a 2 complexity (simple, complex) × 2 grade (6, 7) × 2 hemisphere (left, right) ANOVA for each task. In the addition task, there was a significant main effect of grade (*F*(1, 18) = 6.18, *p* = .023, $\eta_{p}^{2}$ = .256) indicating that there was a change in the parieto-temporal region from deactivation in grade 6 to activation in grade 7 (cf. Fig. 4a). Furthermore, the addition task revealed a significant interaction effect of complexity and hemisphere (*F*(1, 18) = 5.25, *p* = .034, $\eta_{p}^{2}$ = .226) and a post hoc test revealed that only left parieto-temporal activation was higher for simple compared to complex problems (*p* = .037; cf. Fig. 4b). In the multiplication task, there was a significant main effect of grade (*F*(1, 18) = 5.31, *p* = .033, $\eta_{p}^{2}$ = .228) indicating that there was a change in the parieto-temporal region from deactivation in grade 6 to activation in grade 7 (cf. Fig. 4c). No other effects were significant (all *p*s > .05).

To summarize, grade effects were found for a reduction in frontal activation in subtraction and an increase in parieto-temporal activation in addition and multiplication, inverse arithmetic complexity effects were found for parietal activation in multiplication and left parieto-temporal activation in addition, and lateralization effects^2^ were found for frontal activation in subtraction and multiplication and for parietal activation in subtraction, multiplication and division.

#### Additional analysis of fNIRS data

Covariance analyses were conducted in order to account for the behavioral effects of grade and arithmetic complexity. Despite considering the behavioral effect, the effect of grade for parieto-temporal activation in the multiplication task was still significant (*F*(1, 17) = 5.90, *p* = .027, $\eta_{p}^{2}$ = .258) and in the addition task still marginal significant (*F*(1, 17) = 3.26, *p* = .089, $\eta_{p}^{2}$ = .161). On the other hand, the effect of grade for frontal activation in the subtraction task (*F*(1, 17) = 2.27, *p* = .150, $\eta_{p}^{2}$ = .118), the effect of arithmetic complexity for parietal activation in the multiplication task (*F*(1, 17) = 1.07, *p* = .317, $\eta_{p}^{2}$ = .059), and for left parieto-temporal activation in the addition task (*F*(1, 17) = .46, *p* = .506, $\eta_{p}^{2}$ = .026) did not reach significance when considering the behavioral effect.

## Discussion

By investigating the neural underpinnings of calculation in adolescents, we observed activation within the bilateral fronto-parietal network for all basic arithmetic operations. Consistent with the general idea of a fronto-parietal shift with age and experience, the results provide further evidence for a reduction in activation of MFG/IFG from grades 6 to 7 for subtraction and a change in activation of AG/MTG for addition and multiplication. The activation of the left AG/MTG during addition was additionally modulated by arithmetic complexity. Potentially owing to the self-paced design, activation of SPL/IPL was found to be higher for simple than for complex multiplication, reflecting an inverse effect of arithmetic complexity.

In general, activation of the bilateral fronto-parietal network was found for all basic arithmetic operations. Overall, our findings are in line with previous studies showing that parietal regions (i.e., the SPL, IPL, and particularly the IPS) are associated with arithmetic in adults [1, 62, 63]. Furthermore, it seems possible to generalize findings from exact addition in children and adolescents [5, 7, 45, 64] to arithmetic processing for all four basic operations. The present findings further corroborate previous studies [6, 65] which have observed overlapping frontal activation for all basic arithmetic operations in the MFG, IFG, and SFG. Taken together, the results show that adolescents rely on the bilateral fronto-parietal network of arithmetic processing to use the basic arithmetic operations in a natural setting. This activation pattern, however, is influenced by arithmetic development and complexity.

### Developmental activation changes in arithmetic

During arithmetic development, brain activation for arithmetic processing is thought to rely less on frontal and more on parietal areas [11, 16]. From grade 6 to grade 7, the adolescents showed improved subtraction performance which was accompanied by reduced frontal activation in MFG/IFG. This result confirms decreasing frontal activation during development, as suggested by previous cross-sectional [11], longitudinal [18] and training data [12]. In line with these findings, children rely more on domain-general supportive frontal areas such as the IFG for working memory and cognitive control compared to adults [66]. Decreasing frontal activation was not observed for arithmetic operations other than subtraction, possibly because there was no general behavioral improvement in these operations from grade 6 to grade 7. In sum, the current data provide partial support for the developmental fronto-parietal shift during secondary school, since a reduction in frontal activation from grade 6 to grade 7 was found for subtraction, but not for other operations.

Contrary to predictions based on findings comparing children and adolescents to adults [9, 7, 11], no activation increase was observed within the SPL/IPL in any operation over the course of 1 year. Since explicit instruction and training for the basic arithmetic operations concludes earlier in elementary school education (in grade 4), adolescents are presumably proficient in arithmetic, and do not particularly practice or improve on these skills between grades 6 and 7. In light of the conflicting findings on changes in parietal activation during elementary school [17, 18, 32], the maturation of domain-specific processes might be related mostly to initial progress in learning arithmetic and less to general experience with numbers. Thus, the parietal activation increase during arithmetic development might occur earlier than in secondary school when arithmetic knowledge is already established.

In addition to the results for frontal and parietal brain regions, a change from AG/MTG deactivation in grade 6 to increased activation in grade 7 was observed for addition and multiplication. This resembles the finding from Rosenberg-Lee et al. [17] that right AG deactivation in grade 2 changed to above baseline activation in grade 3 for single-digit addition (see also [32]). Considering the role of the AG in the default mode network [67], deactivation in the AG most likely reflects increased task demands for arithmetic processing [68–70]. The same task may therefore become less demanding during development—similar to the way that task demands (and deactivation in the left AG) decline with increasing math competence in adults ([70–72]; for a review see [73]). Although behavioral improvement was only found for simple addition but not multiplication, the grade-related activation change in the AG/MTG might be related to the maturation of arithmetic fact retrieval processes [4]. Altogether, the current data show that arithmetic development during secondary school is to a certain extent accompanied by a reduction in frontal domain-general processing, but does not rely on increased parietal magnitude processing.

Regarding the findings for developmental changes in arithmetic, a note of caution is due since the probe placement varies with changes in head size during development. This is because the distance between the optodes is fixed and the probeset was oriented at parietal sites so that the position of frontal optodes in particular changes with head size. Head size increases by about 0.5 cm during 1 year in this age range (see [74]; see also Table 1 in [75] based on the data of [76]), corresponding to a deviation of 1 mm for the most frontal optodes. This deviation might have affected the frontal results for arithmetic development to some extent, but might be negligible, because fNIRS has a spatial resolution of 3 cm and brain weight does not substantially change between the age of 10 and 14 (see [77]). Thus, longitudinal research reflects a challenge for neuroimaging research.

### Influence of complexity on arithmetic processing in adolescents

The neural correlates of arithmetic processing in adolescents vary with complexity. For instance, activation in the left AG/MTG was higher for simple compared to complex addition blocks, likely because the adolescents solved more simple than complex addition problems in a given length of time. This result corroborates previous findings on the problem size effect showing larger activation of the AG and superior temporal gyri for small problems [5, 30], which likely reflects the retrieval of exact arithmetic facts during single-digit addition problems (cf. [30, 78]). Interestingly, the left AG and MTG are known to belong to the network underlying verbally mediated arithmetic fact retrieval [4, 79]. In the current study, two-digit addition problems without carry procedure were used in the simple condition, so that the activation increase of the left AG/MTG for simple problems reflects separate arithmetic fact retrieval for the sum of the units and the sum of the decades (cf. [80]). Furthermore, the different activation levels of the left AG/MTG associated with arithmetic complexity indicate the increased task demand for complex compared to simple addition problems [67], because regions in the default mode network are generally less active when the task gets more complex [81].

Behaviorally, the carry/borrow effect in addition/subtraction and the problem size effect (comparing two-digit to single-digit problems) in multiplication/division increased task difficulty. However, surprisingly, increased arithmetic complexity was not found to be associated with increased frontal or parietal activation as previously observed (cf. [17, 30, 33, 72]). On the one hand, the self-paced block design might obscure these effects because activation may have increased not only due to difficulty in complex blocks but also due to the larger number of solved problems in simple blocks. It should be noted, however, that fixed-paced block designs or event-related designs are also problematic as arithmetic complexity is confounded with different durations for solving simple and complex problems, which can lead to more extensive activation for complex problems (cf. [82]) and to additional task-irrelevant activation (cf. [83]). On the other hand, the difference between simple and complex blocks in the present study might be too minor to be detected on the neural level, due to design specifications including a balanced problem size, the mix of carry/borrow and no-carry/borrow problems in complex blocks in addition and subtraction, as well as overlapping ranges of the problem size in multiplication and division (different from [33, 72]). Altogether, the specific design as well as properties of the stimulus material seem to have a crucial impact on the dependence of fronto-parietal activation on arithmetic complexity.

### Number magnitude processing in a self-paced block design

Regarding arithmetic complexity, simple multiplication problems elicited larger parietal activation than complex multiplication problems. This inverse effect, in which decreased activation is associated with increased arithmetic complexity, might again be explained by the self-paced block design used here, since it was no longer significant when the number of presented problems was considered as a covariate. More problems were solved during simple than complex blocks, because the solutions for single-digit problems can be faster and relatively automatically retrieved from memory, while two-digit problems mostly need to be solved by slower procedural strategies. Notably, the activation increase in SPL/IPL including the IPS, associated with automatized number magnitude processing [4, 84], was larger for simple blocks, i.e., when more problems were solved and thus elicited increased number magnitude processing. On the contrary, the question arises whether the higher parietal activation usually observed for more complex problems (e.g., [71]) is really due to the calculation procedures underlying their solution or rather due to the longer processing duration (cf. [83]). In sum, the function of the parietal cortex might additionally depend on the number of magnitudes to be processed, i.e., the number of arithmetic problems, besides the processing of number magnitude, i.e., problem size.

## Conclusions and perspectives

In conclusion, the neural activation pattern within the fronto-parietal network of arithmetic processing was found to be similar across arithmetic operations, but still undergoes development in 12–4 year-old adolescents. Consistent with previous studies, a reduction in frontal activation was observed during development and arithmetic complexity was associated with reduced AG/MTG activation. In contrast to previous studies, however, arithmetic complexity elicited *less* parietal activation. We have argued that the current study differed from previous designs by using a self-paced written production paradigm, in which the complexity factor might be confounded with number of trials. Nevertheless, we wish to point out that the inverse arithmetic complexity effect observed in the current study is not just an artifact of the experimental design, but rather reflects the brain activation of adolescents in a natural setting.

More generally, we believe that this study shows that fNIRS seems suitable as an ecologically valid complementary method, especially for research in educational neuroscience [12], because arithmetic processes can be examined in a scholastic setting, where adolescents can solve arithmetic problems in the familiar style of written production (cf. [45, 46]; for a review see [85]).

## Additional file

**Additional file 1: Figure S1.** Cortical activation in the addition task depending on arithmetic complexity and grade (*t* maps). **Figure S2.** Cortical activation in the subtraction task depending on arithmetic complexity and grade (*t* maps). **Figure S3.** Cortical activation in the multiplication task depending on arithmetic complexity and grade (*t* maps). **Figure S4.** Cortical activation in the division task depending on arithmetic complexity and grade (*t* maps). **Figure S5.** Brain-behavior-correlations for the effect of arithmetic complexity in the addition task.

## Footnotes

1 The behavioral analysis is mainly based on the number of presented trials, since this reflects the most appropriate measure for self-paced written responses. The results for RT and ACC analyses should be regarded with caution, since the evaluation of the written responses was not objective due to technical problems with the touch monitor during the production paradigm. Because of these technical problems, the RT and ACC data of one subject is missing for grade 6.

2 Note that the lateralization effects got significant for all operations and the corresponding effect sizes were stronger when the left-handed participants were excluded from analyses (Addition: *F*(1, 15) = 5.44, *p* = .034, $\eta_{p}^{2}$ = .266; Subtraction: *F*(1, 15) = 13.59, *p* = .002, $\eta_{p}^{2}$ = .475; Multiplication: *F*(1, 15) = 18.06, *p* = .001, $\eta_{p}^{2}$ = .546; Division: *F*(1, 15) = 10.87, *p* = .005, $\eta_{p}^{2}$ = .420). This issue of handedness and cortical lateralization needs further investigation in future research.
