# Supplementary material for: The neural correlates of mental arithmetic in adolescents: a longitudinal fNIRS study
Source: Behav Brain Funct. 2018 Mar 10;14:5. doi: 10.1186/s12993-018-0137-8 (PMC5845230; doi:10.1186/s12993-018-0137-8)
Supplement: Supplementary file 1 — Additional file 1: Figure S1. Cortical activation in the addition task depending on arithmetic complexity and grade (t maps). Figure S2. Cortical activation in the subtraction task depending on arithmetic complexity and grade (t maps). Figure S3. Cortical activation in the multiplication task depending on arithmetic complexity and grade (t maps). Figure S4. Cortical activation in the division task depending on arithmetic complexity and grade (t maps). Figure S5. Brain-behavior-correlations for the effect of arithmetic complexity in the addition task. [file 12993_2018_137_MOESM1_ESM.pdf]

ADDITIONAL FILE

Paper

The neural correlates of mental arithmetic in adolescents: A longitudinal fNIRS study

Authors

Christina Artemenko, Mojtaba Soltanlou, Ann-Christine Ehlis, Hans-Christoph Nuerk, Thomas Dresler

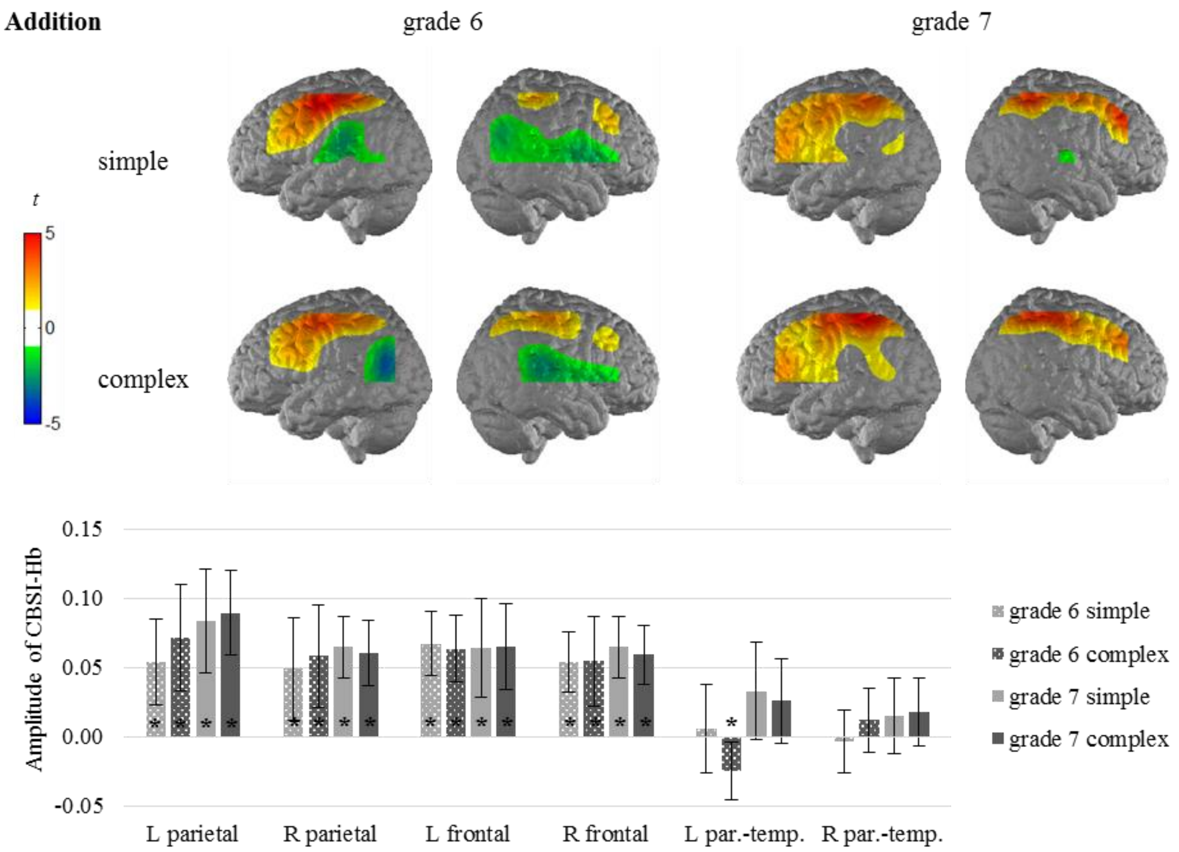

Figure S1: Cortical activation in the addition task depending on arithmetic complexity and grade (*t* maps). Mean amplitudes of activation are shown for each ROI and error bars indicate confidence intervals calculated with respect to normal distribution (* FDR-corrected $p < .05$). Abbreviations: L – left; par.-temp. – parieto-temporal; R – right.

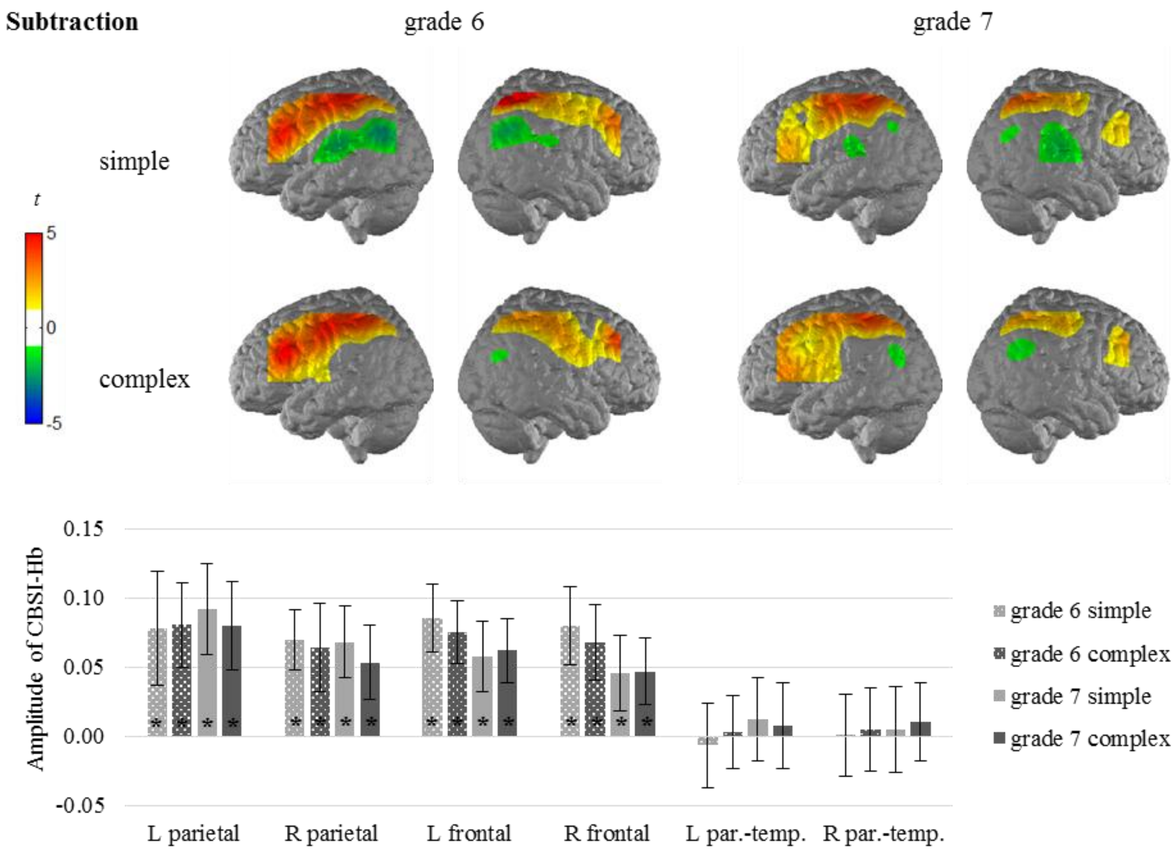

Figure S2: Cortical activation in the subtraction task depending on arithmetic complexity and grade (*t* maps). Mean amplitudes of activation are shown for each ROI and error bars indicate confidence intervals calculated with respect to normal distribution (* FDR-corrected $p < .05$). Abbreviations: L – left; par.-temp. – parieto-temporal; R – right.

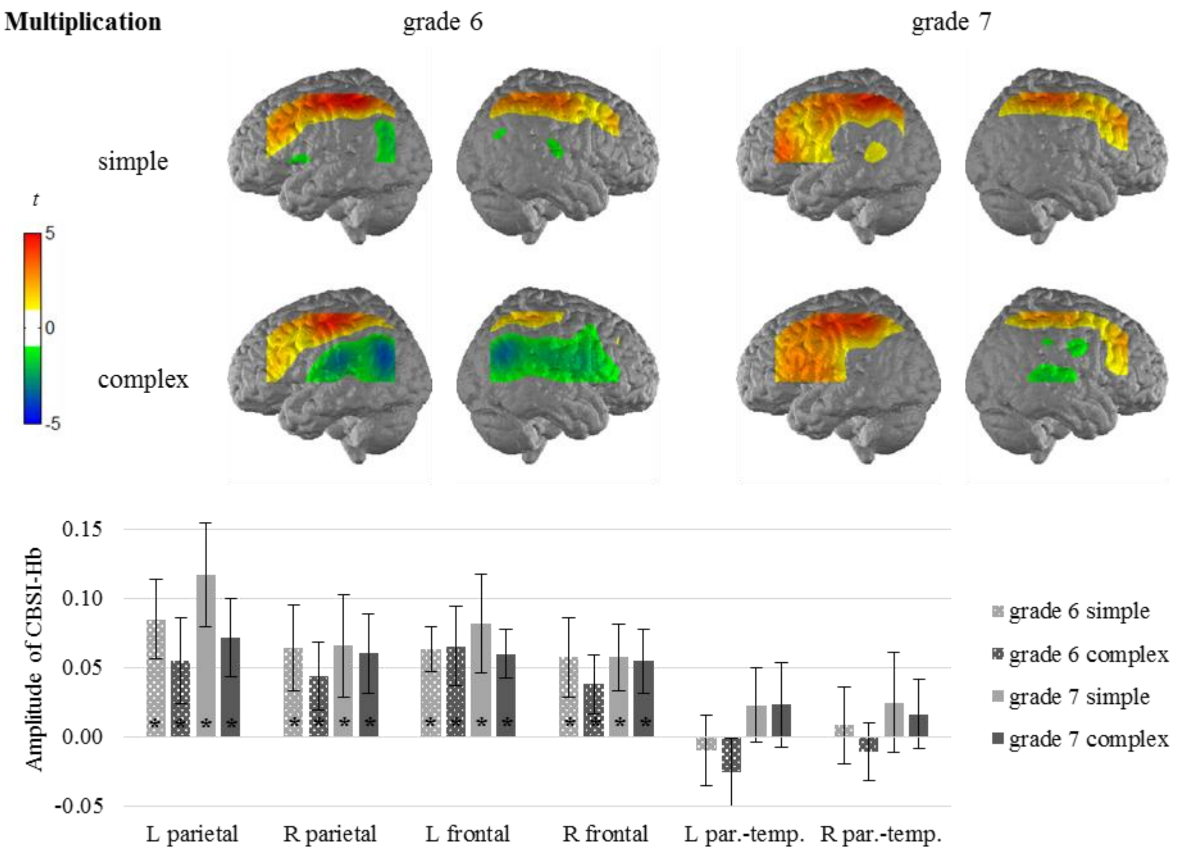

Figure S3: Cortical activation in the multiplication task depending on arithmetic complexity and grade (*t* maps). Mean amplitudes of activation are shown for each ROI and error bars indicate confidence intervals calculated with respect to normal distribution (* FDR-corrected $p < .05$). Abbreviations: L – left; par.-temp. – parieto-temporal; R – right.

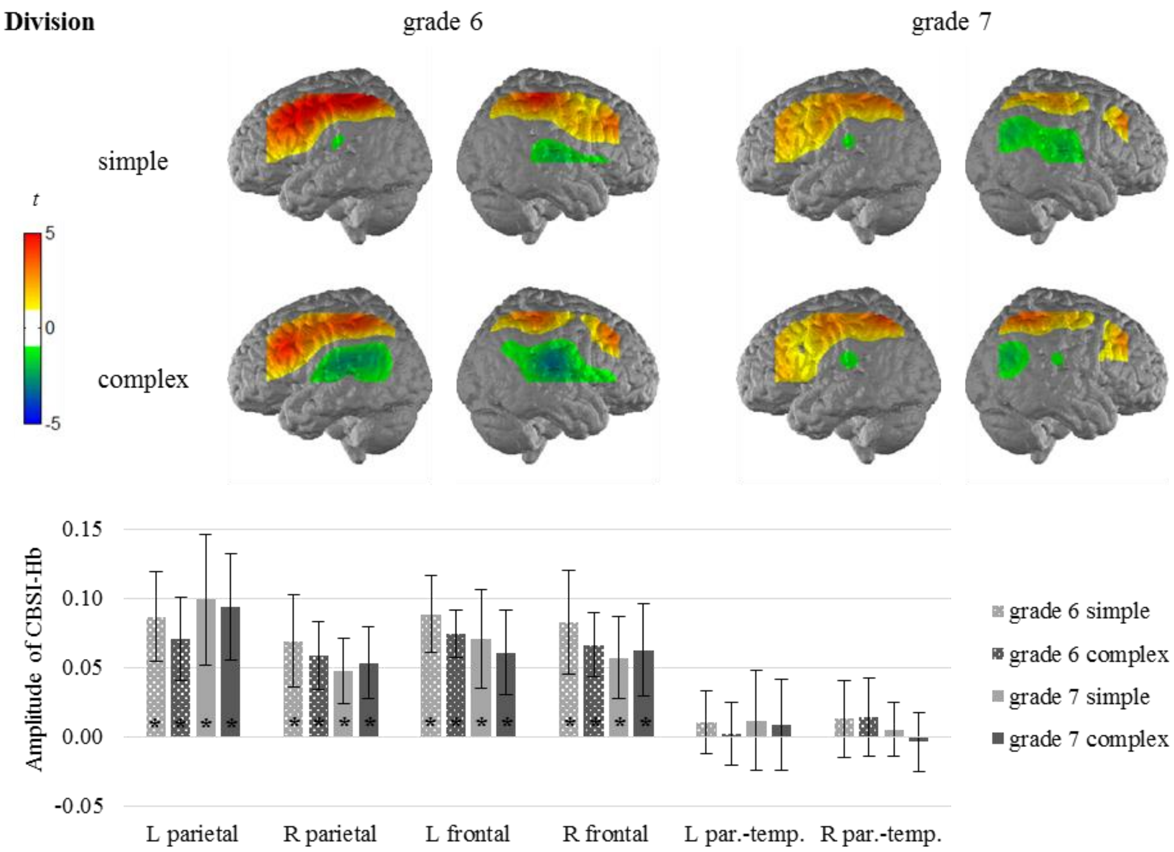

Figure S4: Cortical activation in the division task depending on arithmetic complexity and grade (*t* maps). Mean amplitudes of activation are shown for each ROI and error bars indicate confidence intervals calculated with respect to normal distribution (* FDR-corrected $p < .05$). Abbreviations: L – left; par.-temp. – parieto-temporal; R – right.

Brain-behavior-correlations

Results

Brain-behavior-correlations were calculated for the effects of arithmetic complexity and the effects of grade between the number of presented trials and the cortical activation of each ROI for each task. For the effect of arithmetic complexity in the addition task, a negative correlation was found between the number of presented trials and left frontal activation [r = -.465, p = .045, R^2 = .216] as well as right frontal activation [r = -.488, p = .034, R^2 = .238; cf. Figure S5]. This indicates that with an increasing behavioral effect of arithmetic complexity the cortical effect of arithmetic complexity increases in bilateral frontal areas. Note, however, that the brain-behavior-correlations were not significant after FDR-correction for multiple comparisons. No other correlation was significant [all ps > .05].

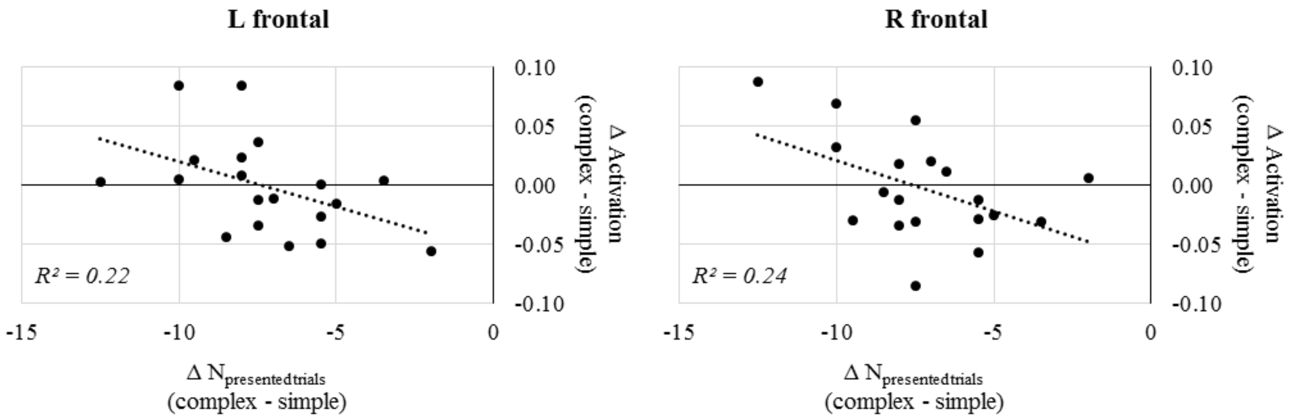

Figure S5: Brain-behavior-correlations for the effect of arithmetic complexity in the addition task. The difference between the number of presented trials for complex vs. simple addition problems was negatively correlated with the difference in cortical activation for complex vs. simple addition problems in the left and right frontal areas. Abbreviations: L – left; R – right.

Discussion

Brain-behavior-correlations were found for the carry effect in addition so that the higher the behavioral carry effect, the higher the neural carry effect for left and right frontal activation (cf. Figure S5, positive activation values). Although this result should be considered cautiously because it was uncorrected, it can be interpreted based on previous research. An increased behavioral carry effect means that the children had a greater challenge with complex problems, which led to higher frontal activation for complex than for simple blocks. This frontal engagement is associated with the carry effect and reflects the higher domain-general demands, like working memory, that are needed to perform the carry procedure, as suggested for the MFG and left IFG by studies in adults (Klein, Willmes, et al., 2010; Klein et al., 2009; Kong et al., 2005; Verner et al., 2013). In addition, for children who revealed a smaller behavioral carry effect, or nearly identical performance on both no-carry and carry problems, the inverse pattern was shown, i.e., higher frontal activation for simple than for complex blocks (cf. Figure S5, negative activation values). This reflects a similar use of frontal resources for no-carry and carry problems, so that activation increases only due to the higher number of problems solved in no-carry blocks. In sum, children might differ in their ability to apply the carry procedure and thus individual differences are reflected in frontal activation for the carry effect.
